# *catena*-Poly[[dichloridozinc(II)]-μ-2,5-di-4-pyridyl-1,3,4-thia­diazole-κ^2^ *N* ^2^:*N* ^5^]

**DOI:** 10.1107/S1600536808001967

**Published:** 2008-01-23

**Authors:** Li-Juan Chen, Ming-Xing Yang, Xiao-Hua Chen, Shen Lin

**Affiliations:** a College of Chemistry and Materials Science, Fuzhou, Fujian 350007, People’s Republic of China

## Abstract

The title compound, [ZnCl_2_(C_12_H_8_N_4_S)]_*n*_, was obtained by crystallization of 2,5-di-4-pyridyl-1,3,4-thia­diazole with ZnCl_2_ in an MeOH/CHCl_3_ solvent system. The structure contains infinite chains of ZnCl_2_ units connected by the bifunctional thia­diazole ligands, with Zn^II^ adopting a distorted tetra­hedral coordination geometry. The dihedral angle between the two pyridyl rings in each ligand is 34.3 (1)°, and the dihedral angles between the thia­diazole ring and the two pyridyl rings are 18.3 (2) and 16.1 (2)°. The shortest Zn⋯Zn distance within each polymeric chain is 11.862 (3) Å, while the shortest inter­chain Zn⋯Zn distance is 7.057 (3) Å.

## Related literature

For related literature, see: Chen *et al.* (2007 ▶); Dong, Ma & Huang (2003 ▶); Dong, Ma, Huang, Guo & Smith (2003 ▶); Du *et al.* (2003 ▶); Fujita (1998 ▶); Huang *et al.* (2004 ▶); Inoue *et al.* (1996 ▶); Maekawam *et al.* (2000 ▶); Moulton & Zaworotko (2001 ▶); Xiong *et al.* (2001 ▶).
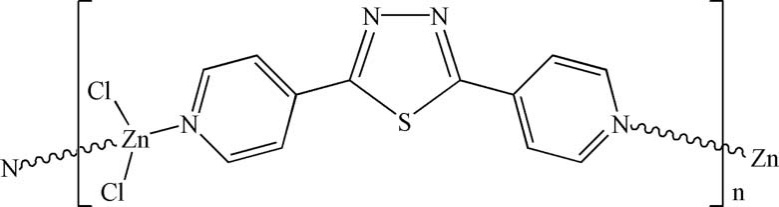


## Experimental

### 

#### Crystal data

[ZnCl_2_(C_12_H_8_N_4_S)]
*M* *_r_* = 376.55
Monoclinic, 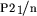

*a* = 12.9990 (13) Å
*b* = 5.4039 (5) Å
*c* = 20.195 (2) Å
β = 101.459 (4)°
*V* = 1390.3 (2) Å^3^
*Z* = 4
Mo *K*α radiation
μ = 2.29 mm^−1^
*T* = 293 (2) K
0.20 × 0.12 × 0.04 mm

#### Data collection

Rigaku Mercury CCD diffractometer
Absorption correction: multi-scan (*CrystalClear*; Rigaku, 2002 ▶) *T*_min_ = 0.690, *T*_max_ = 0.912
10073 measured reflections
3155 independent reflections
2688 reflections with *I* > 2σ(*I*)
*R*_int_ = 0.032

#### Refinement

*R*[*F* ^2^ > 2σ(*F* ^2^)] = 0.037
*wR*(*F* ^2^) = 0.089
*S* = 1.03
3155 reflections
181 parameters
H-atom parameters constrained
Δρ_max_ = 0.43 e Å^−3^
Δρ_min_ = −0.38 e Å^−3^

### 

Data collection: *CrystalClear* (Rigaku, 2002 ▶); cell refinement: *CrystalClear*; data reduction: *CrystalClear*; program(s) used to solve structure: *SHELXS97* (Sheldrick, 2008 ▶); program(s) used to refine structure: *SHELXL97* (Sheldrick, 2008 ▶); molecular graphics: *SHELXL97* and *DIAMOND* (Brandenburg, 1999 ▶); software used to prepare material for publication: *SHELXL97*.

## Supplementary Material

Crystal structure: contains datablocks global, I. DOI: 10.1107/S1600536808001967/bi2274sup1.cif

Structure factors: contains datablocks I. DOI: 10.1107/S1600536808001967/bi2274Isup2.hkl

Additional supplementary materials:  crystallographic information; 3D view; checkCIF report

## Figures and Tables

**Table:** 

Zn1—N4^i^	2.060 (2)
Zn1—N1	2.060 (2)
Zn1—Cl1	2.1939 (8)
Zn1—Cl2	2.2402 (8)

**Table:** 

N4^i^—Zn1—N1	103.11 (9)
N4^i^—Zn1—Cl1	107.44 (6)
N1—Zn1—Cl1	107.78 (7)
N4^i^—Zn1—Cl2	104.00 (7)
N1—Zn1—Cl2	104.64 (6)
Cl1—Zn1—Cl2	127.44 (3)

Symmetry code: (i) 
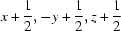
.

**Fig. 1.**
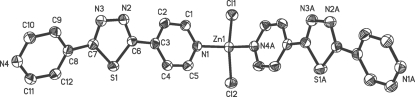
Molecular structure of the title compound showing 30% probability displacement ellipsoids (H atoms are omitted).

## supplementary crystallographic information

### Comment

Much current research activity of supramolecular coordination polymers with novel topologies and structural motifs is driven by their encouraging potential applications in the fields of nonlinear optics, catalysis and separation, magnetism and molecular recognition (Moulton & Zaworotko, 2001, Xiong *et al.*, 2001, Inoue *et al.*, 1996). Therefore, the discovery and rational design of such coordination polymers is an important and very active topic. One useful strategy for the discovery of novel coordination polymers has been the use of various spacer ligands as the building block (Du *et al.*, 2003, Chen *et al.*, 2007). So far, a wide range of infinite frameworks have already been generated with simple 4,4'-bipyridyl-based ligands (Maekawam *et al.*, 2000, Fujita, 1998).

As previously reported (Dong, Ma & Huang, 2003, Huang *et al.*, 2004), oxadiazole-containing ligands can take versatile bonding modes (it can act as mono-, bi-, tri- and even tetradentate ligand) and the structural geometries of the ligands themselves are also variable. On the other hand, d^10^ transition metal-containing complexes often show interesting optical properties. Our interest in the supramolecular coordination polymers lead us to use 2,5-di-4-pyridyl-1,3,4-thiadiazole (*L*) and ZnCl_2_ as precursors to generate the title compound.

Crystallization of *L* and ZnCl_2_ in MeOH/CHCl_3_ mixed solvent system at room temperature yield the title compound, [Zn(C_12_H_8_N_4_S)Cl_2_]_n_. The crystal structure is composed of 1-D zigzag chains with ZnCl_2_ units connected to each other by the N-donor bifunctional ligands. As shown in Fig. 1, the Zn^II^ center adopts a distorted tetrahedral coordination geometry, defined by two N donors from two 2,5-di-4-pyridyl-1,3,4- thiadiazole ligands (*L*) and terminal chloride ligands. Both Zn—N bond distances are 2.060 (2) Å, and the two Zn—Cl bond distances are 2.1938 (8) and 2.2402 (8) Å, respectively. The greatest angular distortion from tetrahedral geometry is exhibited by the Cl—Zn—Cl angle of 127.44 (3) ° and by the N—Zn—N angle of 103.11 (9) °. All these values compare well to those reported in other [Zn(4,4'-bipy)] compounds (Dong, Ma, Huang, Guo *et al.*, 2003). The two pyridyl ring groups in each ligand are not coplanar, and the dihedral angle between them is 34.3 (1) °. The dihedral angles between the thiadiazole ring and the two pyridyl ring groups in each ligand are 18.3 (2) ° and 16.1 (2) °. The intrachain Zn···Zn distance is 11.862 (3) Å, while the shortest interchain Zn···Zn distance is 7.057 (3) Å.

In the crystal, the 1-D zigzag chains stack along crystallographic β axis. In the *ac* plane (Fig. 2), neighboring chains are arranged in opposite directions, and are stacked either in a shoulder-to-shoulder or a staggered fashion.

### Experimental

A solution of ZnCl_2_ (22.7 mg, 0.05 mmol) in MeOH (5 ml) was carefully layered on a solution of 2,5-di-4-pyridyl-1,3,4-thiadiazole (12 mg, 0.05 mmol) in CHCl_3_ (5 ml) in a straight glass tube. After one week, colorless single crystals were obtained (yield *ca* 25% based on Zn).

### Refinement

H atoms were placed geometrically with C—H = 0.93 Å and refined as riding with *U*_iso_(H) = 1.2*U*_eq_(C).

### Crystal data

**Table:**

[ZnCl_2_(C_12_H_8_N_4_S)]	*F*(000) = 752
*M**_r_* = 376.55	*D*_x_ = 1.799 Mg m^−^^3^
Monoclinic, *P*2_1_/*n*	Mo *K*α radiation, λ = 0.71073 Å
Hall symbol: -P 2yn	Cell parameters from 2905 reflections
*a* = 12.9990 (13) Å	θ = 3.9–27.5°
*b* = 5.4039 (5) Å	µ = 2.29 mm^−^^1^
*c* = 20.195 (2) Å	*T* = 293 K
β = 101.459 (4)°	Prism, white
*V* = 1390.3 (2) Å^3^	0.20 × 0.12 × 0.04 mm
*Z* = 4	

### Data collection

**Table:**

Rigaku Mercury CCD diffractometer	3155 independent reflections
Radiation source: fine-focus sealed tube	2688 reflections with *I* > 2σ(*I*)
graphite	*R*_int_ = 0.032
ω scans	θ_max_ = 27.5°, θ_min_ = 3.9°
Absorption correction: multi-scan (*CrystalClear*; Rigaku, 2002)	*h* = −14→16
*T*_min_ = 0.690, *T*_max_ = 0.912	*k* = −7→6
10073 measured reflections	*l* = −26→26

### Refinement

**Table:**

Refinement on *F*^2^	Primary atom site location: structure-invariant direct methods
Least-squares matrix: full	Secondary atom site location: difference Fourier map
*R*[*F*^2^ > 2σ(*F*^2^)] = 0.037	Hydrogen site location: inferred from neighbouring sites
*wR*(*F*^2^) = 0.089	H-atom parameters constrained
*S* = 1.04	*w* = 1/[σ^2^(*F*_o_^2^) + (0.0439*P*)^2^ + 0.6049*P*] where *P* = (*F*_o_^2^ + 2*F*_c_^2^)/3
3155 reflections	(Δ/σ)_max_ = 0.001
181 parameters	Δρ_max_ = 0.43 e Å^−^^3^
0 restraints	Δρ_min_ = −0.38 e Å^−^^3^

### Special details

**Table:**

Geometry. All e.s.d.'s (except the e.s.d. in the dihedral angle between two l.s. planes) are estimated using the full covariance matrix. The cell e.s.d.'s are taken into account individually in the estimation of e.s.d.'s in distances, angles and torsion angles; correlations between e.s.d.'s in cell parameters are only used when they are defined by crystal symmetry. An approximate (isotropic) treatment of cell e.s.d.'s is used for estimating e.s.d.'s involving l.s. planes.
Refinement. Refinement of *F*^2^ against ALL reflections. The weighted *R*-factor *wR* and goodness of fit *S* are based on *F*^2^, conventional *R*-factors *R* are based on *F*, with *F* set to zero for negative *F*^2^. The threshold expression of *F*^2^ > σ(*F*^2^) is used only for calculating *R*-factors(gt) *etc*. and is not relevant to the choice of reflections for refinement. *R*-factors based on *F*^2^ are statistically about twice as large as those based on *F*, and *R*- factors based on ALL data will be even larger.

### Fractional atomic coordinates and isotropic or equivalent isotropic displacement parameters (Å^2^)

**Table:**

	*x*	*y*	*z*	*U*_iso_*/*U*_eq_	
Zn1	1.45602 (2)	1.18961 (6)	0.680619 (15)	0.03305 (12)	
S1	1.23784 (6)	0.24683 (15)	0.42060 (4)	0.04195 (19)	
Cl1	1.34567 (6)	1.43843 (14)	0.71674 (4)	0.04576 (19)	
Cl2	1.59540 (6)	1.29522 (15)	0.63700 (4)	0.0476 (2)	
N1	1.37010 (17)	0.9563 (4)	0.60991 (10)	0.0325 (5)	
N2	1.10437 (18)	0.3373 (5)	0.49394 (11)	0.0374 (5)	
N3	1.06015 (18)	0.1586 (5)	0.45020 (12)	0.0385 (6)	
N4	1.01683 (17)	−0.4543 (4)	0.25889 (10)	0.0320 (5)	
C1	1.2665 (2)	0.9276 (5)	0.60620 (14)	0.0376 (6)	
H1A	1.2329	1.0288	0.6325	0.045*	
C2	1.2081 (2)	0.7546 (6)	0.56503 (14)	0.0370 (6)	
H2A	1.1362	0.7423	0.5630	0.044*	
C3	1.2576 (2)	0.5981 (5)	0.52646 (12)	0.0321 (6)	
C4	1.3644 (2)	0.6283 (6)	0.53025 (14)	0.0397 (7)	
H4A	1.4002	0.5273	0.5052	0.048*	
C5	1.4169 (2)	0.8089 (6)	0.57142 (14)	0.0403 (7)	
H5A	1.4883	0.8300	0.5727	0.048*	
C6	1.1964 (2)	0.4066 (5)	0.48470 (12)	0.0308 (6)	
C7	1.1201 (2)	0.0891 (5)	0.40919 (13)	0.0314 (6)	
C8	1.0874 (2)	−0.1003 (5)	0.35760 (13)	0.0317 (6)	
C9	1.0054 (2)	−0.2613 (5)	0.36310 (14)	0.0360 (6)	
H9A	0.9736	−0.2536	0.4004	0.043*	
C10	0.9723 (2)	−0.4310 (5)	0.31285 (13)	0.0364 (6)	
H10A	0.9165	−0.5344	0.3165	0.044*	
C11	1.0960 (2)	−0.3019 (5)	0.25406 (14)	0.0390 (7)	
H11A	1.1278	−0.3178	0.2169	0.047*	
C12	1.1329 (2)	−0.1232 (6)	0.30118 (14)	0.0389 (6)	
H12A	1.1875	−0.0193	0.2954	0.047*	

### Atomic displacement parameters (Å^2^)

**Table:**

	*U*^11^	*U*^22^	*U*^33^	*U*^12^	*U*^13^	*U*^23^
Zn1	0.03407 (19)	0.0322 (2)	0.03165 (17)	−0.00116 (13)	0.00357 (13)	0.00049 (13)
S1	0.0336 (4)	0.0522 (5)	0.0429 (4)	−0.0112 (3)	0.0145 (3)	−0.0163 (3)
Cl1	0.0474 (4)	0.0385 (4)	0.0521 (4)	0.0064 (3)	0.0115 (3)	−0.0035 (3)
Cl2	0.0436 (4)	0.0520 (5)	0.0500 (4)	−0.0069 (3)	0.0159 (3)	0.0043 (4)
N1	0.0314 (11)	0.0336 (13)	0.0313 (11)	0.0002 (9)	0.0032 (9)	−0.0022 (10)
N2	0.0351 (12)	0.0412 (14)	0.0370 (12)	−0.0051 (10)	0.0094 (10)	−0.0086 (11)
N3	0.0344 (12)	0.0436 (14)	0.0377 (12)	−0.0044 (11)	0.0075 (10)	−0.0077 (11)
N4	0.0343 (12)	0.0300 (12)	0.0309 (10)	0.0007 (9)	0.0045 (9)	0.0001 (9)
C1	0.0335 (14)	0.0388 (16)	0.0396 (14)	0.0016 (12)	0.0054 (12)	−0.0069 (13)
C2	0.0287 (14)	0.0399 (16)	0.0415 (15)	0.0031 (12)	0.0048 (12)	−0.0050 (13)
C3	0.0323 (13)	0.0339 (15)	0.0287 (12)	−0.0026 (11)	0.0024 (10)	0.0031 (11)
C4	0.0347 (15)	0.0472 (17)	0.0382 (14)	−0.0017 (13)	0.0096 (12)	−0.0131 (13)
C5	0.0291 (14)	0.0494 (19)	0.0424 (15)	−0.0044 (12)	0.0069 (12)	−0.0086 (14)
C6	0.0298 (13)	0.0323 (14)	0.0302 (12)	0.0016 (11)	0.0056 (10)	−0.0008 (11)
C7	0.0306 (13)	0.0315 (14)	0.0318 (12)	−0.0011 (11)	0.0054 (11)	0.0022 (11)
C8	0.0300 (13)	0.0326 (14)	0.0307 (12)	0.0009 (11)	0.0012 (10)	−0.0016 (11)
C9	0.0380 (15)	0.0404 (16)	0.0315 (13)	−0.0054 (12)	0.0119 (12)	−0.0001 (12)
C10	0.0379 (15)	0.0356 (16)	0.0365 (14)	−0.0072 (12)	0.0097 (12)	0.0005 (12)
C11	0.0371 (15)	0.0463 (18)	0.0361 (14)	−0.0061 (13)	0.0134 (12)	−0.0080 (13)
C12	0.0322 (14)	0.0443 (16)	0.0416 (15)	−0.0082 (13)	0.0106 (12)	−0.0040 (13)

### Geometric parameters (Å, °)

**Table:**

Zn1—N4^i^	2.060 (2)	C2—H2A	0.930
Zn1—N1	2.060 (2)	C3—C4	1.384 (4)
Zn1—Cl1	2.1939 (8)	C3—C6	1.466 (4)
Zn1—Cl2	2.2402 (8)	C4—C5	1.373 (4)
S1—C7	1.727 (3)	C4—H4A	0.930
S1—C6	1.729 (3)	C5—H5A	0.930
N1—C5	1.341 (3)	C7—C8	1.462 (4)
N1—C1	1.343 (3)	C8—C12	1.390 (4)
N2—C6	1.302 (3)	C8—C9	1.397 (4)
N2—N3	1.357 (3)	C9—C10	1.372 (4)
N3—C7	1.301 (3)	C9—H9A	0.930
N4—C10	1.337 (3)	C10—H10A	0.930
N4—C11	1.337 (3)	C11—C12	1.374 (4)
C1—C2	1.375 (4)	C11—H11A	0.930
C1—H1A	0.930	C12—H12A	0.930
C2—C3	1.391 (4)		
			
N4^i^—Zn1—N1	103.11 (9)	C3—C4—H4A	120.3
N4^i^—Zn1—Cl1	107.44 (6)	N1—C5—C4	123.0 (3)
N1—Zn1—Cl1	107.78 (7)	N1—C5—H5A	118.5
N4^i^—Zn1—Cl2	104.00 (7)	C4—C5—H5A	118.5
N1—Zn1—Cl2	104.64 (6)	N2—C6—C3	122.0 (2)
Cl1—Zn1—Cl2	127.44 (3)	N2—C6—S1	113.3 (2)
C7—S1—C6	86.95 (13)	C3—C6—S1	124.68 (19)
C5—N1—C1	117.7 (2)	N3—C7—C8	122.0 (2)
C5—N1—Zn1	121.31 (18)	N3—C7—S1	113.6 (2)
C1—N1—Zn1	120.60 (18)	C8—C7—S1	124.45 (19)
C6—N2—N3	113.2 (2)	C12—C8—C9	117.7 (3)
C7—N3—N2	113.0 (2)	C12—C8—C7	122.4 (2)
C10—N4—C11	117.7 (2)	C9—C8—C7	119.9 (2)
C10—N4—Zn1^ii^	121.41 (18)	C10—C9—C8	119.3 (2)
C11—N4—Zn1^ii^	120.52 (18)	C10—C9—H9A	120.3
N1—C1—C2	122.6 (3)	C8—C9—H9A	120.3
N1—C1—H1A	118.7	N4—C10—C9	122.9 (3)
C2—C1—H1A	118.7	N4—C10—H10A	118.6
C1—C2—C3	119.4 (3)	C9—C10—H10A	118.6
C1—C2—H2A	120.3	N4—C11—C12	123.4 (2)
C3—C2—H2A	120.3	N4—C11—H11A	118.3
C2—C3—C4	117.9 (3)	C12—C11—H11A	118.3
C2—C3—C6	119.5 (2)	C11—C12—C8	118.9 (3)
C4—C3—C6	122.6 (2)	C11—C12—H12A	120.5
C5—C4—C3	119.3 (3)	C8—C12—H12A	120.5
C5—C4—H4A	120.3		

Symmetry codes: (i) *x*+1/2, −*y*+1/2, *z*+1/2; (ii) *x*−1/2, −*y*+1/2, *z*−1/2.
